# Teachers’ Dispositions Toward Mindfulness in EFL/ESL Classrooms in Teacher-Student Interpersonal Relationships

**DOI:** 10.3389/fpsyg.2021.754998

**Published:** 2021-09-16

**Authors:** Xiaochuan Song, Xuan He

**Affiliations:** ^1^School of Foreign Studies, Henan Agricultural University, Zhengzhou, China; ^2^School of Foreign Languages, Shaanxi Normal University, Xi’an, China

**Keywords:** psychological factors, English as a foreign language, English as a second language, mindfulness, emotional factors

## Abstract

The psychological factors of English as a foreign language (EFL) and English as a second language (ESL) teachers have significant roles in any language learning context. Previous studies in the related literature have shown that L2 learners’ learning, psychological factors, and emotional factors are closely related to teachers’ psychological factors. Mindfulness as one of the psychological attributes of L2 teachers and as a complex and multi-faceted construct influences l2 teachers’ professional development. Hence, this study aims to review the notion of mindfulness and its role in L2 teaching as a profession and pave a way for further research, highlighting its indispensable role in teacher-student relationships. To achieve this goal, this study has reviewed the theoretical perspectives of mindfulness, the construct of mindful L2 teaching education, and mindfulness as a closely related concept to teacher wellbeing. Based on the findings, some pedagogical implications for the policymakers, teacher trainers, materials developers, L2 teachers, and learners are provided. Finally, directions for future research are suggested to the interested L2 researchers.

## Introduction

The psychological factors of L2 teachers have significant roles in any L2 learning context and affect language teaching as a profession. Hence, L2 learners’ learning, psychological factors including affective and emotional factors are closely related to teachers’ psychological factors. Teaching has been considered a stressful and challenging profession that bears responsibility on teachers’ shoulders. To tackle these psychological barriers, teachers need to be empowered with influential psychological factors. Positive psychology as a new area of psychology emerged to conceptualize the positive side of psychological factors (Dewaele et al., 2019), including but not limited to emotions, care, wellbeing, credibility, resilience, and mindfulness of teachers. Instead of focusing on negative psychological factors, positive psychology has focused on the positive sides of psychological factors which have been considered as a paradigm shift from learner-centered pedagogy (Seligman et al., 2006; MacIntyre et al., 2016). Positive psychology has rooted in the humanistic approach of psychology and its aim is to investigate the effect of emotions and feelings on peoples’ quality of life. Oxford (2016) argued that while the focus of positive psychology is on the wellbeing and positive sides of human psychological factors, it does not neglect abnormalities and adversities of human psychological factors. In the domain of positive psychology, although there have been potential advantages of mindfulness, little attention has been dedicated to teachers’ mindfulness as one of the components of teachers’ wellbeing (Yuan et al., 2020; Wang et al., 2021). Little attention has been paid to the influence of mindfulness, as a complex and multi-faceted construct, on L2 teachers’ professional development and teacher-student interpersonal factors (Xie and Derakhshan, 2021). Hence, this study aims to review the notion of mindfulness and its role in L2 teaching as a profession and pave a way for further research.

## Mindfulness: A Theoretical Perspective

Over the past decades, mindfulness has emerged in educational psychology and teacher education followed by the flourishing body of research. Although the interest in mindfulness has grown recently, there is no consensus on the definition of the term. Even one problem with the research studies on mindfulness relates to the issue that there have been some studies (e.g., Jennings et al., 2011) that explained to practice mindfulness but failed to give an explicit definition for that. Mindfulness has been viewed by researchers (Langer, 1997; Li, 2021) as a conscious awareness of instant comprehensible experience. Langer (1997) represented mindfulness as a process involving understanding problems and solutions of daily life experience. In this sense, one can focus on a specific mental state instantly while gaining a deeper perspective on what is happening. As a pioneer of the mindfulness field, Kabat-Zinn (2003, p. 145) defines mindfulness as “a state or quality of mind that arises through paying attention on purpose, in the present moment, and nonjudgmentally to the unfolding of experience moment by moment.” Mindfulness can be considered as a process rather than a product of activity; it is dynamic rather than static since it focuses on ongoing life experiences. Awareness of moment-to-moment experiences without making a judgment about these experiences is a key concept in the definitions of mindfulness. Brown and Ryan (2003) consider mindfulness awareness of present events and experiences which can be received by attention. Mindfulness, as a complex and idiosyncratic construct, can be considered a concept consisting of several elements. It includes personal and professional features which help teachers to connect with different features of life experiences (Yuan et al., 2020). Yuan et al. (2020) found some critical dimensions constructed to the concept of mindfulness including time, change, self, teaching and learning practices, professional development, and the context of L2 teaching. In summary, based on the above definitions, we can conceptualize mindfulness as conscious awareness of moment-to-moment life experiences, which is purposeful, focused, and non-judgmental.

## Mindful L2 Teaching Education

L2 teacher education has been designed to grow L2 teachers’ professional development. Johnson and Golombek (2016) in their insightful book *Mindful L2 Teacher Education* looking at the teachers’ professional development *via* a sociocultural perspective argued that L2 teacher education embeds culturally and socially educational practices. To be mindful, teachers need to know themselves, their affective and cognitive factors, and their desired utopia. Mindful L2 teacher education as a process involves teachers focus on the learning experiences, the nature of mediation between teacher and teacher trainer, shaping a new understanding, and teachers’ attentive interactions with learners. Furthermore, “teachers need to be mindful of the consequences of our pedagogy on how teachers come to understand both the scope and impact of their teaching” (Johnson and Golombek, 2016, p. 164). In other words, teachers need to be conscious about their motivation, orientations, and intentions when practicing their pedagogy. Mindfulness is also a paradigm shift against dualistic thinking which has been the dominant philosophy in the Aristotelian school of thought (Johnson and Golombek, 2016). The concentration of this philosophy is on dualisms, such as mind and body, theory and practice, and expert and inexpert. In contrast, dialectic thinking focuses on the connections and interrelationships of these constructs, process, dynamicity, and change which is in line with the mindfulness training movement. In this sense, as Johnson and Golombek (2016) stated, mindfulness helps teacher to think dialectically so they can reflect on their future based on their present and past. On the other hand, there have been some efforts to move mindfulness away from L2 pedagogy. For instance, the pre-designed curriculum that needs teachers to be content-oriented tells to teachers what to do, what to say, and even what to contemplate regardless of the context of teaching. Mindful L2 teacher education has been considered a movement against this discourse in the sense that it focuses on the present moment while considering core values, purposes, and identities in pedagogy. In these cases, mindfulness may play a significant role in assisting learners and teachers to be positive when facing challenging situations to promote teachers’ and learners’ professional development.

Mindfulness can be taught and learned. Mindfulness training includes various activities expanding from daily practices to intensive training courses. Mindfulness training has been well established in clinical and health contexts (Brown and Ryan, 2003) and it has been shown that mindfulness training has a positive effect on affective and psychological factors, such as wellbeing, stress, mood, and attention (Baer et al., 2006; Lutz et al., 2008; Li, 2021). Sharp and Jennings (2016) argued that teachers’ mindfulness helps to foster the formation of positive relationships with students, impact their psychological factors, and grow their professional development which may lead to student’s higher achievement. Mindful practices can help learners to overcome their distractions and keep their focus on their surroundings. In the classroom context, mindfulness helps teachers to be aware of their inactive pedagogical capabilities so they can aware of what is happening around them. Furthermore, mindfulness practices also help teachers to enhance their understanding of the body (such as fatigue), mind (thinking capacity), and feeling (nervousness; Bernay, 2014). Jennings and Greenberg (2009) argued that mindfulness helps teachers to be positive and strong in challenging situations which in turn lead to learners’ academic achievement. Furthermore, Schussler et al. (2016) suggested that practicing mindfulness increases teachers’ professional performance. By practicing, mindfulness and making it a tangible habit and tangible and long-lasting advantages for learners and teachers can be ensured (Altan et al., 2019).

## Mindfulness and Teacher Wellbeing

Wellbeing of teachers, as a rudimentary construct of positive psychology, has been defined as teachers’ judgment and salinification levels of his/her mental and physical condition. A number of studies (e.g., Baer et al., 2006; Hue and Lau, 2015) show that mindfulness training activities are effective in improving teachers’ psychological health (such as teachers’ wellbeing) and decreasing teachers’ stress and burnout, and these effects mostly have been investigated through subjective first-person measures. Hwang et al. (2017) considers teachers’ wellbeing and their performance as the consequences of mindfulness training. Fathi and Derakhshan (2019) state that psychological wellbeing helps teachers to establish a positive relationship with their students which lead to higher academic performance. It is also a useful construct leading to higher job satisfaction (Kidger et al., 2016) and increased learners’ performance. In a systematic review, Hwang et al. (2017) investigated 16 research conducted up to 2015 in mindfulness. The results suggested that few studies have been conducted to investigate mindfulness-based intervention, and many of those studies have focused on teachers’ mindfulness and wellbeing and different interpretations of mindfulness and its influences were taken into account to describe mindfulness-based interventions. Furthermore, Chiesa et al. (2011) investigated the mindfulness and psychological wellbeing of teachers. The results showed that mindfulness training increased psychological capacities and regulation strategies which in turn lead to increased sustainability. Learners also are affected by mindfulness-based training reported that teachers’ chronic stress and burnout may lead to learner-teacher relationships which negatively affect learners’ performance. Furthermore, a number of studies have shown that mindfulness has positively related to learners’ academic achievement, task performance, emotion regulation, and learning productivity (Kee and Liu, 2011; Flook et al., 2013; Rosenstreich and Margalit, 2015).

## Pedagogical Implications

The results of such a study which investigates teachers’ psychological attributes (e.g., mindfulness) can boost the awareness of learners, teacher trainers, policymakers, material designers, and other researchers in the field. Derakhshan (2021) describes the implications for teachers to be with language teaching as a demanding profession that involves adversities, tension, and traumatic experiences. Teacher trainers also can enhance their knowledge of mindfulness-related strategies which in turn lead to competent teachers. Material developers also can be benefited in the sense that they should consider the psychological attributes of teachers which lead to a less stressful and defensive context of learning. Mindful teaching also has some implications for students in a way that they can be aware of their responsibility in the learning process. In this sense, classroom tensions are removed and a relaxed classroom atmosphere can be created and learners’ motivation and involvement can be enhanced. Finally, the results of mindfulness-based studies result are useful for policymakers so they can have a deeper understanding of teaching values and teacher psychological and affective factors.

## Conclusion and Directions for Future Research

Mindfulness-based training has recently got the interest of many educators in L2 learning and teaching. The review suggests that mindfulness-based training is capable of reducing learners’ and teachers’ anxiety, helping learners to be attentive learners, and be critical thinkers. Although most of the conducted studies in the related literature have focused on mindfulness and its positive effects on different psychological dimensions, the effect of implementing mindfulness techniques on the development of different language skills has rarely been investigated in the related literature. Furthermore, most of the conducted studies in the field of mindfulness have been explored by the use of quantitative cross-sectional studies. Conducting longitudinal mindful-based training studies which investigate the long-term effect of implementing such programs on teachers’ and learners’ performance and considers the dynamic aspect of the issue can be another avenue of further research. Moreover, conducting mixed-method research that looks at the concept of mindfulness from different qualitative and quantitative perspectives can deepen our understanding of the contributions of mindfulness and its dimensions on L2 teacher professional development. Needless to say, learners play a significant role in any L2 learning context. However, in comparison with the studies conducted on L2 teacher education and mindfulness, there have been few studies investigating mindful-based interventions on the side of language learners as well as the role of mindful learners and teachers in interpersonal relationships between teachers and students. Since contextual, cultural, and demographic factors play an important role in any education setting in general and language learning in particular, conducting mindfulness-based training studies which take into account contextually oriented factors are of significant importance. It has been argued that learners’ academic performance, motivation, and attitude all depend on teachers’ affective factors, hence investigating the interplay of teacher mindfulness and learners’ mindfulness can be fruitful in understanding the pedagogical benefits of mindfulness-based teaching and learning.

## Author Contributions

XS drafted the first manuscript and XH revised the original version. Both of the two authors agreed on the order of the authors before they got this final draft ready for submission to Frontiers in Psychology. All authors contributed to the article and approved the submitted version.

## Conflict of Interest

The authors declare that the research was conducted in the absence of any commercial or financial relationships that could be construed as a potential conflict of interest.

## Publisher’s Note

All claims expressed in this article are solely those of the authors and do not necessarily represent those of their affiliated organizations, or those of the publisher, the editors and the reviewers. Any product that may be evaluated in this article, or claim that may be made by its manufacturer, is not guaranteed or endorsed by the publisher.
